# The First 20 Hours of Geopolymerization: An in Situ WAXS Study of Flyash-Based Geopolymers

**DOI:** 10.3390/ma9070552

**Published:** 2016-07-08

**Authors:** Ross P. Williams, Arie van Riessen

**Affiliations:** John de Laeter Centre, Curtin University of Technology, GPO Box U1987, Perth WA 6845, Australia; a.vanriessen@curtin.edu.au

**Keywords:** geopolymers, flyash, dissolution, XRD, time-resolved, amorphous

## Abstract

This study followed the first 20 h of flyash geopolymerization at 70 °C using time resolved Wide Angle X-ray Scattering (WAXS). The extent of dissolution of the amorphous phase of the flyash was determined to range from 29% to 54% for the different formulations trialed. The dissolution rate of the flyash significantly reduced after the first 5 h for all samples. During the formation stage of the geopolymer there were significant temporal variations in the chemistry of the dissolved solution due to the rate of flyash dissolution, with a relative standard deviation of 20%, 57% and 24% for the Si/Al, Na/Al and H/Si ratios, respectively. Utilizing the Power Law, scattering in the low angle region of the WAXS pattern combined with the geopolymer peak area yielded a measure which correlated with the compressive strength—providing a new method to measure the flyash dissolution and geopolymer formation processes independently. The evolution of several zeolite-like phases was followed, noting there are different formation mechanisms involved even within the same sample. Four samples were examined with compressive strengths ranging from 14(2)–50(9) MPa, each was synthesized with flyash from Collie Power Station (Western Australia) activated with sodium silicate solution of varying concentrations.

## 1. Introduction

### Geopolymer Kinetics

Utilizing X-ray diffraction, the growth/dissolution kinetics of the crystalline phases can be readily studied [1,2]. However, the amorphous (non-crystalline) phases can also be quantified [3], allowing kinetics of amorphous phase(s) dissolution/growth to be quantified [4,5].

There are several reported studies on the kinetics of geopolymerization, utilizing energy dispersive X-ray diffraction [1], Attenuated Total Reflection Fourier Transform Infrared spectroscopy (ATR-FTIR) [6], differential scanning calorimetry (DSC) [7] and AC Impedance Spectroscopy (ACIS) [8]. Although in situ Small Angle X-ray Scattering (SAXS) and Wide Angle X-ray Scattering (WAXS) has been applied to track zeolite formation by many authors [9,10,11,12,13], in situ SAXS and WAXS has only been applied in a limited way to the early kinetics studies of metakaolin geopolymers [14].

De Silva et al. [15], in a study of metakaolin geopolymer kinetics, found that setting times increased with increasing Si/Al until Si/Al = 2, then decreased with further increases to Si/Al. Zhang et al. [16,17] found in a Isothermal Conduction Calorimetry (ICC) study of sodium hydroxide and sodium silicate activated metakaolin geopolymer that the extent of reaction increased with Na/Al between 0.74 to 1.47, but also increased the zeolite or zeolite-like phase crystallization rates. They also found that increases in curing temperature from 25 to 40 °C increased the rate of reaction and the extent of reaction, the sensitivity of extent of reaction to temperature was less for sodium hydroxide activated samples with a Na/Al > 1. However, for sodium silicate activated samples they found the extent of reaction was maximized at an intermediate temperature (35 °C). A sample cured at 40 °C had a higher extent of reaction in the first 5 h, but reached a plateau, leading to the useful conclusion that the fast formation of geopolymer can, in fact, hinder the dissolution process, thus decreasing the overall extent of reaction.

Jozić et al. [14] studied kinetics of metakaolin geopolymer with in situ SAXS and WAXS. The geopolymer material studied appears to have a composition dissimilar to other geopolymers discussed in this study, the composition was Si/Al = 1.6 and 2.1; Na/Al = 0.9 and 1.72 and H/Si = 1.34 and 2.1; the key difference being the low H/Si, whereas typical values are 4–7 for structural geopolymers. This coupled with the electron micrograph of the microstructure showing a sparsely agglomerated material, which is more consistent with a very weak geopolymer. The finding of Jozić et al. [14] for two geopolymer samples were that the fractal dimension of the geopolymer reduced from 2.9 to 2.4 after about 4 h at 70 °C for one sample and reduced from 3.3 to 2.3 after about 8 h for another sample.

Steins et al. [18] studied ageing of metakaolin geopolymer with ex situ SAXS, Ultra Small Angle X-ray Scattering (USAXS), Small Angle Neutron Scattering (SANS) and nitrogen sorption. Steins et al. found that a fraction of the porosity transformed from open to closed porosity, and, after about one month, the water in the pores dehydrates and is replaced by air.

Studying well cured metakaolin geopolymers, Maitland et al. [19] found that USANS/SANS was predominantly from Power law scattering from open pores, and the scatter was insensitive to geopolymer composition. In addition to the SANS and USANS data, Maitland et al. provided electron microscopy data to support the bimodal classification of the pores, designated level 1 and level 2 pores. The level 2 pores are <10 nm pores, also observed by others with TEM [20,21,22]. Steins et al. [18], utilizing SANS and Brunauer, Emmett and Teller (BET) method, found that the pore volume decreased over a six-month time scale for both sodium and potassium silicate activated metakaolin. Additionally, Steins et al. [18] found the pore size distribution range was 5–20 nm for sodium silicate activated, and 2.5–8.5 nm for potassium silicate activated, metakaolin after six months. Phair et al. [23] applied USANS to alkali activated flyash and slags, however, the size scale probed was on the order of 500–5000 nm, which is essentially probing the size and shape of agglomerates of the smaller subunits rather than the subunits themselves.

## 2. Materials and Methods

### 2.1. Geopolymer Synthesis

The flyash used in this study was from Collie Power station, Collie, Western Australia. The flyash used was representatively sampled from the same 20 kg samples characterized in a previous study [24]. The amorphous SiO_2_ and Al_2_O_3_ contents of this flyash were 32(3) wt % and 16(2) wt %, respectively of a total of 64(4) wt % amorphous. The geopolymer compositions were chosen to be within uncertainties of those for which compressive strength data were collected in a previous study [25]. The compositions and compressive strengths are summarized in Table 1, only the amorphous component of the flyash is considered in the formulation, the crystalline species were considered inert. These samples were cured at 70 °C for 24 h.

The geopolymers were formulated for the matrix compositions in Table 1. The amorphous composition of the flyash was used to formulate the mixtures to produce 10 g of sample, the other starting materials were sodium silicate solution (Grade D, PQ Australia Pty Ltd., Dandenong, VT, Australia); sodium hydroxide (Chem-Supply, Gillman, SA, Australia, AR grade) and deionized water.

The solutions were mixed together and allowed to rest at ambient temperature overnight in a 25 mL polypropylene vial. The flyash was quickly added to this vial and mixed at high speed using a mixing head on a Dremel rotary tool for 1 min. The sample slurry was then sucked into a 100 mm long section of PEEK tubing using a 1 mL syringe for suction. The ends of the tubing were then sealed using 1.6 mm diameter steel nails. The tubes of samples were then placed in an ice bucket until 6 samples were prepared, then the samples were loaded into the 35 position sample changer of the WAXS instrument with temperature set at 25 °C. Initial patterns were collected before the capillary temperature was increased to 70 °C, as measured by a type-K thermocouple placed inside PEEK tubing loaded as a dummy sample in the sample changer.

### 2.2. WAXS Data Collection

The in situ experiment was conducted on the SAXS/WAXS beamline at the Australian Synchrotron (Melbourne, VT, Australia); essentially the beam line has an in-vacuum undulator, double crystal monochromators and K-B (Kirkpatrick-Baez) focusing mirrors. A 20.000 keV photon beam was focused on a ~100 µm symmetrical spot. A MAR-165 camera (marXperts GmbH, Norderstedt, Germany) was used to measure the patterns at a camera length of approximately 231 mm, with the camera placed such that the beam center was near the edge of the detector to maximize the angular range collected. Using these conditions, the measured useful angular range was 1° to 25° 2θ, which is equivalent to a *q*-range of approximately 0.18 Å^−1^ to 4.4 Å^−1^ or equivalent to 2.5° to 65° 2θ with copper Kα radiation.

Selecting the correct capillaries for this experiment was challenging. The capillary needs to be resistant to concentrated sodium hydroxide solution at 70 °C for 24 h, as well as being available as thin tubing with an appropriate wall thickness to allow high X-ray transmission. Polyetheretherketone (PEEK) was found to meet these requirements. PEEK is highly resistant to hot caustic solutions, has a low atomic number and is available as a 1.59 mm diameter capillary with 0.01 mm wall thickness (Supelco Analytical, Bellefonte, PA, USA).

### 2.3. WAXS Data Processing

**Figure 1:**
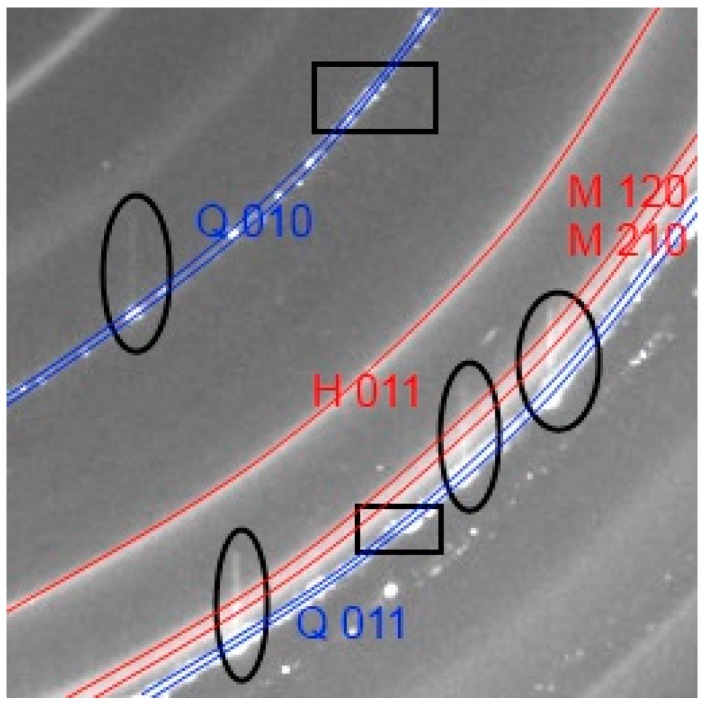
Cropped view of the 2D WAXS pattern from a geopolymer sample. The circled annotations on the image show the pixel overflow in the columns where a large quartz (Q) crystal saturated the detector, overflowing into adjacent pixels. This effect was reduced by applying a masking process to remove saturated pixels. The square annotations show the quartz peaks where it is clear there is a distribution of quartz d-spacings. Note, the hematite (H 011) and mullite (M 120 and M 210) peaks are smooth continuous rings, indicating the crystallite size is small compared to the diffraction information volume, hence, there are thousands to millions of crystallites.

The exact values of camera length and camera tilt were obtained by using Fit2d (V12.012, European Synchrotron Radiation Facility (ESRF), Grenoble, France) to refine a pattern of LaB_6_ (SRM 660b, National Institute of Standards and Technology (NIST), Gaithersburg, MD, USA). The 2D diffraction datasets for each geopolymer sample were filtered using a script in MATLAB (The Mathworks, Natick, MA, USA). The 2D diffraction patterns exhibited the expected sharp ring patterns from the mullite, secondary quartz and crystalline iron oxides (Figure 1). The patterns also had superimposed multiple spot patterns from the much larger crystallite size of primary quartz [24], a number of which saturated the detector. The absolute intensity of any point on the ring is low compared to the intensity of primary quartz spot patterns, which are produced by only a relatively small number of very large crystallites (10–50 µm). The spots were found by thresholding intensity values above 4000 units, then expanding the size of each cluster of high intensity pixels by convolving a circle of 27 pixels diameter to generate a mask, which was used to set all affected pixels to an intensity of 65,535. The patterns were normalized to the incident beam intensity; corrected for the transmission factor of each sample and the PEEK capillary ‘blank’ was subtracted. To account for the measured transmission factor of each sample, an empty capillary was used to subtract the capillary diffraction signal.

The filtered and normalized patterns were loaded into Fit2d, where the threshold value to mask out saturated pixels was set at 65,534, and then radially averaged applying the calibration for camera length and tilt. The filtering process improved the smoothness of the quartz peak intensity versus time.

### 2.4. WAXS Phase Identification

A MATLAB scriptwas used to visualise the diffraction patterns as a function of time using 2D color plots; these were used to select 3 to 5 representative diffraction patterns to perform phase search/match. The search/match was conducted using EVA 15.0 (Bruker-AXS, Karlsruhe, Germany) with the Powder Diffraction File database (PDF 4+ (2009)) [26]. The phases identified were exported such that accurate phase markers could be displayed on the MATLAB output. The identified phases were used to find the most suitable crystal structures for use in the subsequent Rietveld refinements using the Inorganic Crystal Structure Database (ICSD) (2010/1) [27], PDF 4+ (2009) and American Mineralogist crystal structure databases [28].

### 2.5. WAXS Data Analysis

A model was refined to the data using TOPAS 4.2 (Bruker-AXS, Karlsruhe, Germany), in launch (script) mode. The Bragg peaks from crystalline phases were modelled using the crystal structures (i.e., Rietveld method), whereas the diffuse scattering from the amorphous phases were modelled using a split pseudo Voigt (SPV) function per phase. The intended refinement method attempted is referred to as a parametric refinement [29,30], that is to say, all the patterns (per sample) were simultaneously opened and many parameters were linked together between patterns to reduce the number of refined parameters using analytical functions with a few refinable parameters. However, selecting the analytical functions to get a good fit of the data was not achieved, so the more typical and basic method of setting global variables for some parameters was required. The refined parameters are summarized in Supplementary Materials Table S1, The instrument resolution function (IRF) was modelled using a convolution model described by Müller et al. [30], refined with the LaB_6_ (NIST SRM 660b) data.

Refining a common peak position and shape for the amorphous materials in the flyash and a common peak position shape for the geopolymer allows the degree of flyash dissolution to be determined using the area ratio of the geopolymer and flyash peaks [3]. This also allows the amorphous peak position of the geopolymer to be determined; the method was shown to be more accurate than using the measured shift in a single peak.

The “background” was modelled as required using Equation (1), where X is 2θ; and a, b, c and d are refinable parameters. The c parameter from herein will be referred to as the Power Law Pre-factor.

(1) $$\left(X\right)=a+\text{bX}+\frac{c}{X^{d}}$$

The phase concentrations can be presented on an absolute scale with mullite being used as an ‘internal standard’ to the calibrate system. The mullite concentration in the flyash was determined previously [24] where it was demonstrated that mullite did not react significantly in any of the samples making it a suitable internal standard. It was noted that the mullite patterns exhibited small variations of periodic nature which correlated with other phases indicating that the use of an internal standard was suitable.

The Rietveld analysis was conducted with TOPAS in launch mode using the ‘conserve-memory’ directive to allow refinement with all ~500 datasets simultaneously.

The relative concentrations of geopolymer (sodium aluminosilicate gel), flyash (amorphous part) and the Power Law pre-factor were calculated by normalising each signal, such that the range was between 0% and 100%.

### 2.6. SAXS Data Collection

The same capillaries were sent back to the SAXS/WAXS beamline and the beamline scientist, Nigel Kirby, generously collected SAXS data from the same sample capillaries 1 month after the WAXS experiments. The SAXS pattern was collected on the Pilatus 1M (DECTRIS Ltd., Baden-Daettwil, Switzerland) detector with beam energy of 15.0 keV and a camera length of approximately 1576 mm. The data was collected at 2 s and 5 s exposure times. Data were collected at ambient pressure and temperature. The data collection range was approximately 0.01–0.7 Å^−1^.

## 3. Results

### 3.1. Preliminary Analysis of WAXS Data

Visual inspection of how the 2D diffraction patterns change with time (not shown) reveals a ring pattern with sharp rings and diffuse rings, which are analogous to Bragg peaks and diffuse peaks from amorphous phases in conventional XRD patterns, both slowly varying as the reaction proceeds. The sharp rings were from zeolite-like phases which form suddenly after an induction time for the sample CFA-1.8-0.8-5.5, the zeolite-like phases in the other samples formed more gradually. The diffuse rings from the amorphous phases gradually increase in diameter. The sharp ring patterns from the mullite, iron oxides and newly formed zeolite-like phases are radially homogeneous, indicating a very small crystallite size relative to the diffraction information volume, i.e., thousands to millions of crystallites in the beam.

Superimposed on the rings are primary quartz spot patterns that move in position from pattern to pattern for a period of time and then stop moving. During the first part of the reaction the primary quartz spots randomly appear and disappear. The radius of the spots is slightly larger than the ring pattern of the secondary quartz, which is consistent with the previous findings that there are two populations of quartz in the flyash [24]. These spots cause a data processing challenge as the mean intensity is very imprecise because there are not a large number of crystals contributing to the spot, but can be dealt with, as detailed in Section 2.3.

The search and match results revealed the same phases as previously found in this flyash batch [24], but with the addition of some zeolite or zeolite-like phases that could not be uniquely identified. The phases could not be identified as there were very few peaks and they did not match well with the peak positions for possible phases in the Powder Diffraction File or in specific zeolite collections [31]. The zeolite-like phases found in the geopolymer probably have a high degree of interstitial substitution which has shifted the d-spacing away from the more pure zeolites in the databases.

Visual inspection of the WAXS data time series as 2D color contour images (Supplementary Materials, Figures S1–S4), where intensity shown by color scale, reveals several new crystalline phases which form with time. After an induction period, there was an increase in the low angle (Q < 1 Å^−1^) intensity and a ‘shift’ in the diffuse scattering peak to higher angle. The induction period and reaction rates vary between geopolymer formulations.

**Figure 2:**
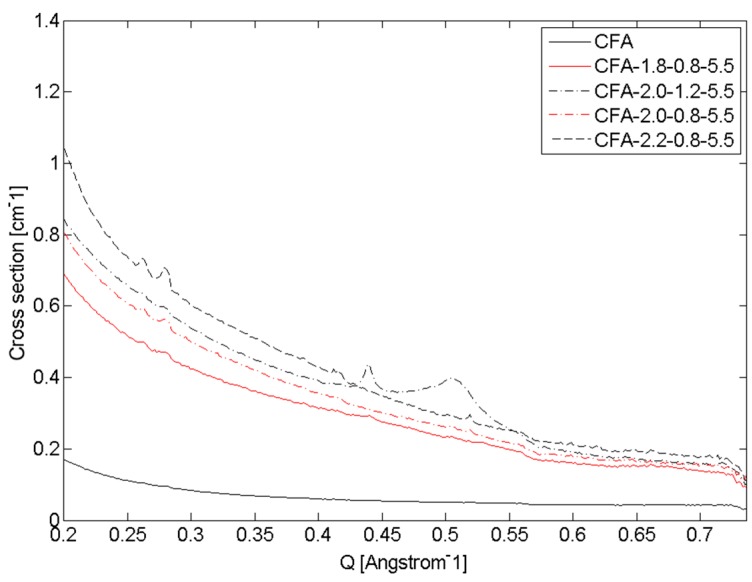
SAXS patterns confirm that the increased intensity at low angle (<3° 2θ) seen in the WAXS data is from small angle scattering phenomenon rather than a broad Bragg peak of a large zeolite-like phase. Collie Flyash (CFA) is also shown a comparison. The useful limit of the WAXS data was Q = 0.18 Å^−1^. Although there are Bragg peaks in the SAXS data, they are not source of the majority of the intensity. Note, cross section is a calibrated representation of intensity.

Using only the WAXS data, it could not be confirmed that the increase in intensity at low angle (Q < 1 Å^−1^) was either broad Bragg peaks from large zeolite-like structures or a small angle scattering phenomenon, hence, additional SAXS data was collected from the same capillaries one month after the initial experiment. The SAXS data in Figure 2 shows that, although there are some Bragg peaks from zeolite-like phases in the low angle region of the WAXS patterns (Q = 0.18 to 0.53 Å^−1^), it is not the significant source of intensity. The ex situ SAXS study of geopolymer aged for one month did not reveal anything significant when compared to the WAXS and/or compressive strength data [25].

**Figure 3:**
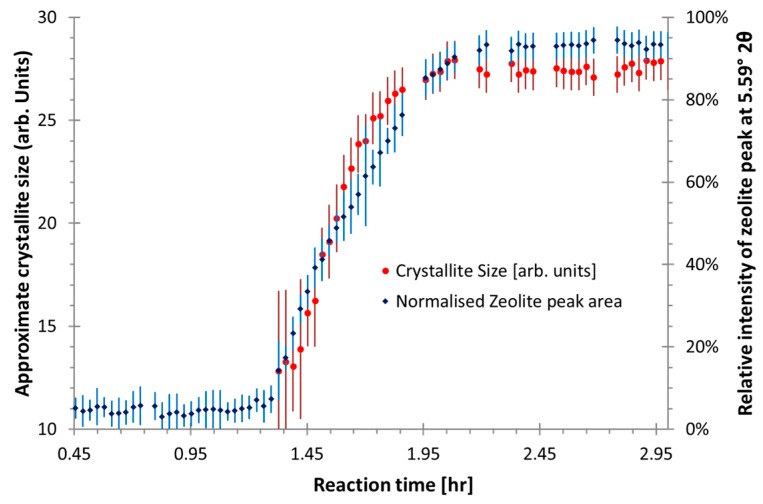
The relative peak area and crystallite size for a zeolite-like phase peak at Q = 1.97 Å^−1^ for sample CFA-1.8-0.8-5.5. The plateau of the crystallite size as the relative peak intensity reach ~90% indicates there is nucleation period like the formation of typical zeolites, but with no crystal growth subsequently. Note, due to a fitting artifact causing crystallite size to randomly fluctuate when relative peak area is <5%, (as there is insufficient intensity to fit the peak) these peaks have been omitted for clarity. The error bars represent 1 estimated standard error from the refinement process.

**Figure 4:**
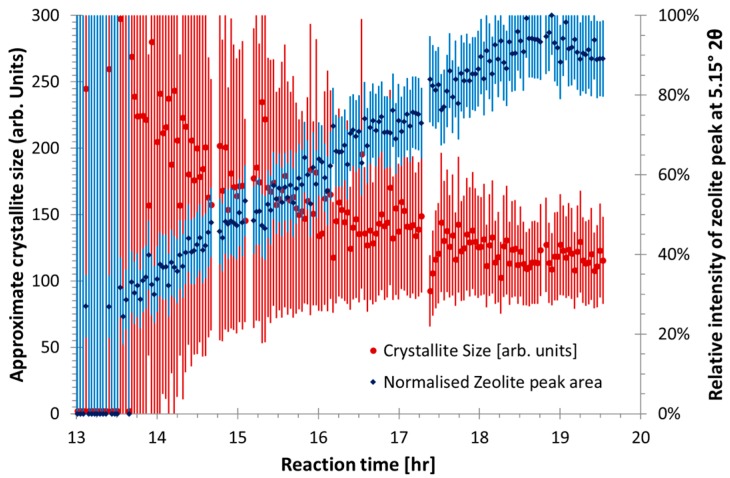
Relative peak area and crystallite sizes of a zeolite-like phase at Q = 1.82 Å^−1^ for sample CFA-1.8-0.8-5.5. The peak is of low intensity compared to the background, hence, the noisy signal. The error bars represent 1 estimated standard error from the refinement process.

Preliminary analysis of the zeolites-like phases were conducted prior to the full quantitative analysis, the peak shapes of the zeolite-like phases were fitted using Lorentzian peaks using TOPAS 4.2. Figure 3 shows peak area and a non-calibrated measure of crystallite size or coherently scattering domain size, by the integral breadth method [32], for the major zeolite-like phase peak (Q = 1.97 Å^−1^) in CFA-1.8-0.8-5.5. It shows that after an induction time, the concentration and crystallite size rapidly increases and plateaus out. This supports the understanding that during the induction time many crystallite seeds form and the phase concentration increases via crystal growth. Conversely, the other unidentified zeolite-like phase in the same sample which has an induction period of around 12 h, Figure 4 shows different formation behavior. The crystallite size instantaneously increases to a plateau value, while the peak intensity is still low (20% of maximum), and then the intensity continues to increase and the crystallite size slightly decreases. This indicates the crystal growth rate is much faster than the nucleation rate for the second zeolite-like phase.

### 3.2. Quantitative Analysis of WAXS Data

**Figure 5:**
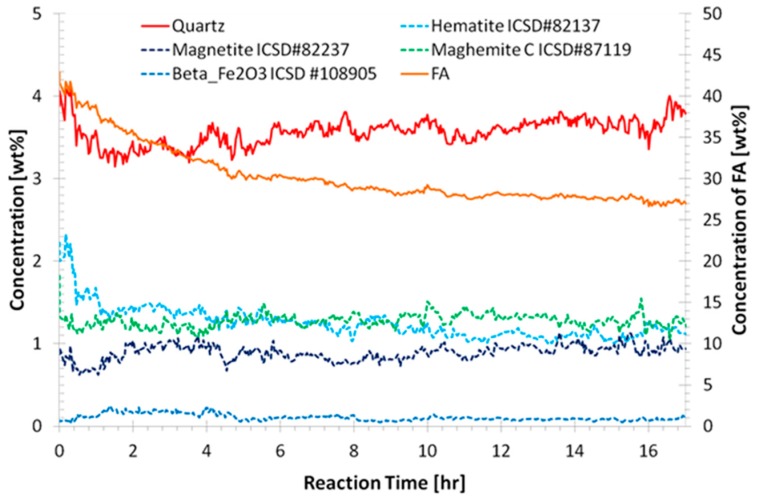
Quantitative phase results from the refinement CFA-2.0-1.2-5.5. The concentration of the flyash (FA) amorphous phase is shown on the right hand side axis. Results from other samples are shown in Supplementary Materials Figure S5.

The quantitative phase results obtained from Rietveld refinement are shown in Figure 5 for CFA-2.0-1.2-5.5 and in Supplementary Materials Figure S5 for all samples. The quartz concentration for all samples is variable, as discussed above. The amorphous flyash content decreased smoothly with time and is summarized in Table 2.

## 4. Discussion

It was found that the extent of flyash amorphous phase dissolution does not correlate with the physical properties. For all samples, the phase quantified as hematite decreased in the first few hours by approximately 50%. It is unlikely that hematite would dissolve in the high pH environment, which indicates that a portion of the quantified hematite is actually a soluble spinel phase with a very similar diffraction pattern. With the current datasets, given the low concentration of the phase (1–2 wt % in the samples) it is not possible to draw stronger conclusions. The peak positions of the amorphous phases are shown in Table 3. The peak positions of the geopolymer amorphous phase are at slightly lower angles than found for metakaolin geopolymers using a similar modelling method [3], which was between 3.15 and 3.22 Å for metakaolin geopolymer compared to 2.92 Å to 3.06 Å for these flyash geopolymers.

Using the extent of dissolution of the flyash amorphous phase the Si/Al, Na/Al and H/Si of the reaction product can be calculated as a function of time; this is made available in Supplementary Materials Figure S6. The composition of geopolymer phase and zeolite-like phase(s) are not explicitly known, hence were not subtracted. The dissolution of the flyash amorphous component was assumed to be congruent. As expected, the elemental concentrations in solution available for geopolymer formation varied significantly during the geopolymer growth phase, as dissolution of the flyash amorphous phase continues during the geopolymer formation. The mean and standard deviation of the elemental ratio are shown in Table 4. The statistics were calculated when the relative geopolymer concentrations were between 10% and 90%.

**Figure 6:**
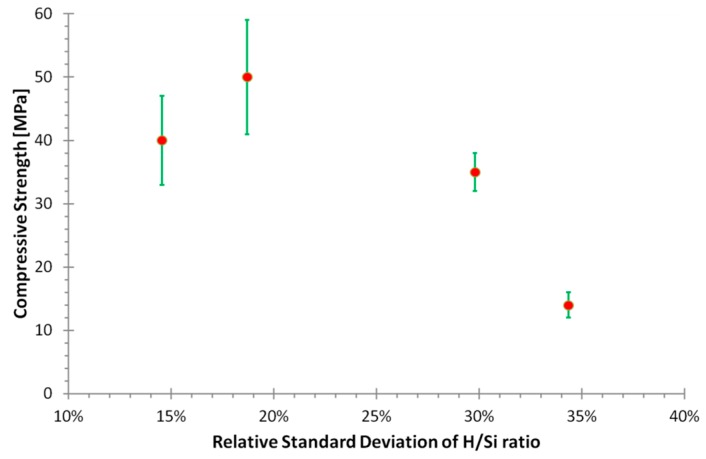
The relative standard deviation of calculated atomic H/Si in solution during geopolymer phase growth (between 10% and 90% of relative geopolymer concentration). Note, the formation of geopolymer and zeolite-like phases was not accounted for and the inverse correlation between compressive strength and the variability of the solution.

The relative standard deviation of H/Si ratio in solution during the formation stage correlates inversely with compressive strength (Figure 6). This correlation indicates strong geopolymer phases may only form when the conditions are within a specific range. The measured parameter which influences the calculated relative standard deviation of the calculated H/Si ratio is the dissolution rate of the amorphous fraction of flyash during the time the geopolymer forming. The results indicate if the flyash is dissolving at a high rate during geopolymer formation the resulting geopolymer will be of lesser strength. The large variation of the solution composition in which geopolymer forms does prompt the question of whether there is a similar variation in the actual composition of the geopolymer matrix. The quantitative EDS work of Rowles and O’Connor [34] of metakaolin geopolymer found the relative standard deviation of Si/Al and Na/Al was orders of magnitude less; for example the matrix composition of the strongest sample was Si/Al = 3.2(1) and Na/Al = 1.36(6), which is a relative standard deviation of 3% and 4%, respectively, compared to mean relative standard deviations in this study of 20% and 56% for Si/Al and Na/Al, respectively.

**Figure 7:**
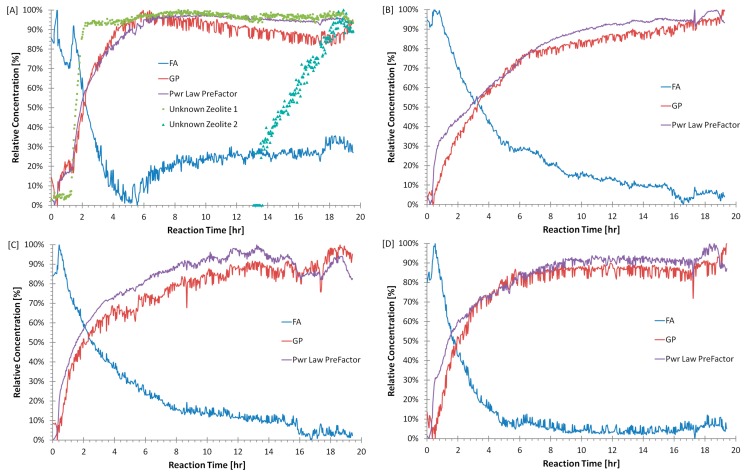
Refined output from WAXS data for samples: (**A**) CFA-1.8-0.8-5.5; (**B**) CFA-2.0-1.2-5.5; (**C**) CFA-2.0-0.8-5.5 (**D**) CFA-2.2-0.8-5.5; the time evolution of the diffuse scattering peaks of flyash (FA) and geopolymer (GP) compared with the Power Law pre factor. Note for plot (**A**), that the induction period for zeolite 1 and geopolymer are approximately equal.

Figure 7 shows the dissolution of the flyash amorphous phase and evolution of the geopolymer amorphous phase and the Power Law pre-factor. Both are displayed as a fraction between their minimum and maximum values. The Power Law pre-factor is correlated to the specific surface area of the sample and in this case indicates the presence of very small species; both the small geopolymer species and other small species resulting from the dissolution of the amorphous fraction of the flyash [35] and the nanoporosity. Once the geopolymer has solidified, surface area would also have a major contribution from the porosity. Quantification of the surface area from the SAXS data was beyond the scope of this study. The relative concentration of flyash in Figure 7a increases after 6 h, this was probably an indication that another amorphous phase with diffuse peak in a similar position to flyash has formed, rather than a geopolymer phase.

**Figure 8:**
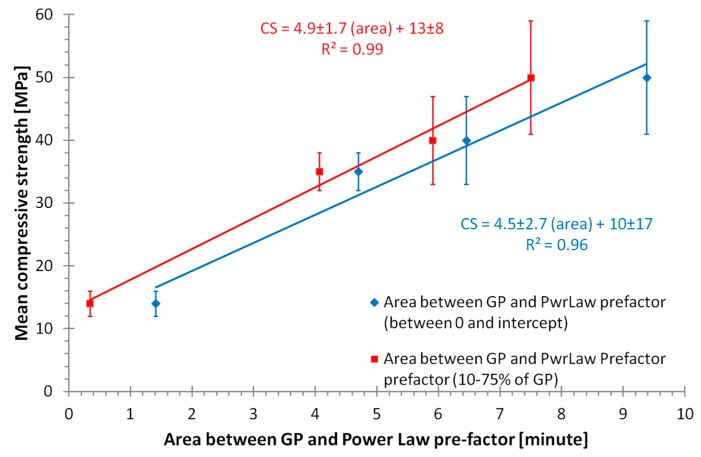
Relationship between the area between geopolymer formation and Power Law pre-factor (WAXS data), as measured by two metrics. This limited data set (*n* = 4) does have a strong positive linear relationship for both methods of determining the area, indicating good correlation between the integrated area and the compressive strength. As described in the text, the area should be correlated to the quantity of flyash dissolved before significant geopolymerization occurs, this supports the findings of Zhang et al. [16,17], that the strength is maximized by prolonging the dissolution stage compared to the start of the geopolymerization.

The area between the curves of the relative intensities of the Power Law pre-factor and geopolymer signal correlates strongly with the mean compressive strength of the geopolymer samples (Figure 8). The area was determined by two methods; the first the area between the geopolymer and Power Law pre-factor from start of the reaction until the first incept of those curves. The second method was the area between the curves for the time between when the geopolymer had a relative concentration of 10% and 75%. The second method provides an improved correlation to the mean compressive strength of the sample. Given the Power Law pre-factor is likely correlated with the sum of dissolved aluminosilicates and geopolymer species; and the geopolymer signal is correlated with the geopolymer species; the area should be correlated to the quantity of flyash dissolved before significant geopolymerization occurs. This supports the findings of Zhang et al. [16,17] that the strength is maximised by prolonging the dissolution stage compared to the start of the geopolymerization. This method has significant value for future experiments as it allows the dissolution and geopolymer formation (polycondensation) steps to be optimised separately. However, although the method would be predictive of strength, the mechanism could also be related to porosity which also effects strength [19].

## 5. Conclusions

In this study, in situ WAXS has been used to follow the reaction of flyash dissolution and subsequent geopolymerization yielding:

- Confirmation that there are multiple mechanisms of zeolite-like phase formation even within the same geopolymer sample.
- A new method has been identified to separately measure the progress of feedstock dissolution and geopolymer formation in situ, which will allow a large number of optimization experiments to be completed in the future.
- Identification of significant temporal variation in the composition of the solution from which the geopolymer forms; the relative standard deviation of the H/Si ratio during formation was correlated to the physical properties.

## Figures and Tables

**Table 1:** Composition and compressive strength (CS) of the geopolymer samples. Calculations of the elemental ratio only consider the amorphous composition of the flyash, i.e., the crystalline component was considered inert and the values indicate the target final composition assuming 100% reaction of the amorphous component of the flyash. The value in the parentheses corresponds to the standard deviation of repeat compressive strength tests.

Sample Name	Si/Al	Na/Al	H/Si	CS (MPa)
CFA-1.8-0.8-5.5	1.8	0.8	5.5	14(2)
CFA-2.0-0.8-5.5	2.0	0.8	5.5	35(3)
CFA-2.0-1.2-5.5	2.0	1.2	5.5	50(9)
CFA-2.2-0.8-5.5	2.2	0.8	5.5	40(7)

**Table 2:** Summary of the flyash amorphous phase concentration; initial concentration was calculated from mix formulation and QPA results of the starting flyash [24] and the final concentration was determined by the refinement of the WAXS data. The Compressive Strength (CS) has been shown again for comparison.

Sample Name	Initial (wt %)	Final (wt %)	Extent of Dissolution (%)	CS (MPa)
CFA-1.8-0.8-5.5	46	33	29	14(2)
CFA-2.0-0.8-5.5	44	20	54	35(3)
CFA-2.0-1.2-5.5	43	27	38	50(9)
CFA-2.2-0.8-5.5	42	24	42	40(7)

**Table 3:** The refined d-spacings of the flyash and geopolymer amorphous phase peaks. The geopolymer amorphous phase peak positions are smaller than found for the metakaolin geopolymer [33] which were between 3.15 and 3.22 Å. The flyash amorphous phase peak position was allowed vary between samples in the refinement.

Sample Name	Flyash Amorphous Phase (Å)	Geopolymer Amorphous Phase (Å)
CFA-1.8-0.8-5.5	3.96	2.92
CFA-2.0-0.8-5.5	4.00	3.02
CFA-2.0-1.2-5.5	4.08	2.99
CFA-2.2-0.8-5.5	4.03	3.06

**Table 4:** The mean atomic ratio in the solution during geopolymer phase growth (between 10% and 90% of relative GP concentration); and the mean final extent ratio. The statistics were weighted by time. The final extent ratios are the mean of the last 15 patterns. Note, the formation of geopolymer and zeolite-like phases was not accounted for as the specific chemistry was not known.

Sample Name	Solution during Reaction			Final Extent		
	Si/Al	Na/Al	H/Si	Si/Al	Na/Al	H/Si
CFA-1.8-0.8-5.5	2.3(4)	4.4(28)	22.5(77)	2.23(2)	3.9(2)	21.6(6)
CFA-2.0-0.8-5.5	2.5(6)	2.2(16)	11.2(33)	2.24(1)	1.46(4)	8.9(2)
CFA-2.0-1.2-5.5	2.8(4)	4.3(17)	13.8(26)	2.51(3)	3.2(1)	11.7(3)
CFA-2.2-0.8-5.5	3.2(8)	2.3(12)	10.8(16)	3.0(1)	2.1(2)	10.4(5)

## Supplementary Materials

The following are available online at www.mdpi.com/1996-1944/9/7/552/s1.
