# Supplementary material for: The First 20 Hours of Geopolymerization: An in Situ WAXS Study of Flyash-Based Geopolymers
Source: Materials (Basel). 2016 Jul 8;9(7):552. doi: 10.3390/ma9070552 (PMC5456921; doi:10.3390/ma9070552)
Supplement: Supplementary file 1 [file materials-09-00552-s001.pdf]

Supplementary Materials: The First 20 Hours of Geopolymerization: An in Situ WAXS Study of Flyash-Based Geopolymers

Ross P. Williams and Arie van Riessen

Table S1. The parameter scheme used in the refinement. Note: GP represents geopolymer and FA flyash. When the parameter is refined in common, it means that the variable are analytically linked such that one variable is refined per pattern or phase, reducing the number of variables refined.

Parameter	Overall		Per Pattern	
	Common	Each Phase	Common	Per Phase
Background	–	–	X	–
Camera length error	X	–	–	–
Phase scale factor	–	–	–	X
Crystallite Size	–	–	–	X
Lattice Parameters	–	X	–	–
GP peak position	X	–	–	–
GP peak shape	X	–	–	–
GP peak intensity	–	–	X	–
FA peak position	X	–	–	–
FA peak shape	X	–	–	–
FA peak intensity	–	–	X	–

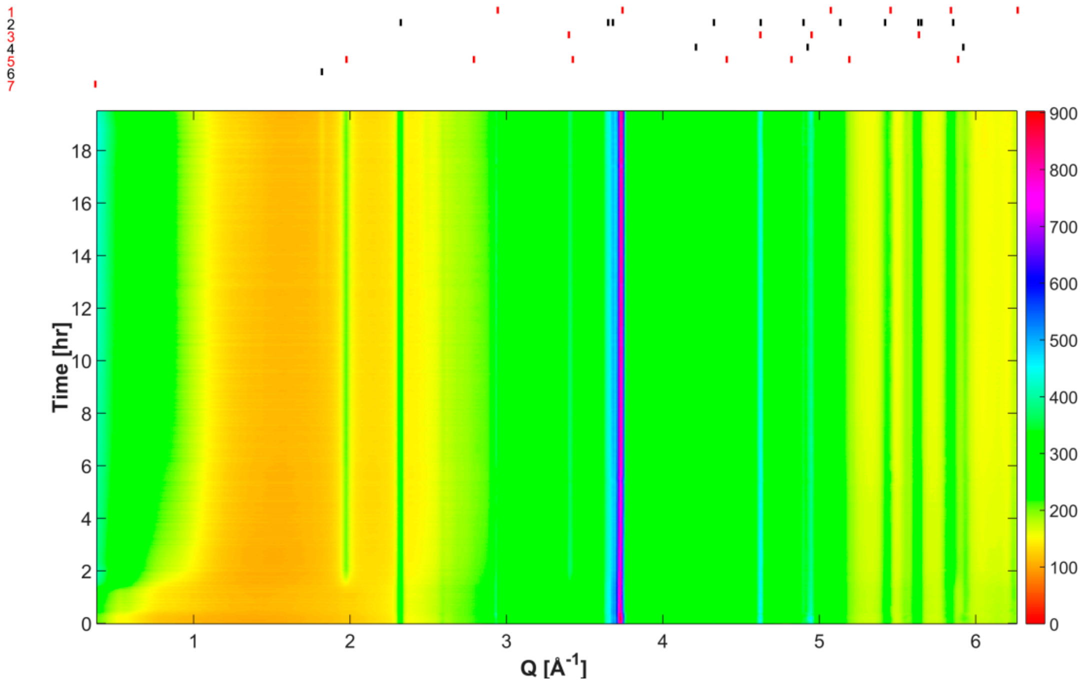

Figure S1. Time resolved diffraction pattern of CFA-1.8-0.8-5.5. Features include the evolution of low angle intensity (Q < 1 Å^{-1}), formation of two zeolite phases and the 'shift' in the amorphous peak position. The peak positions of significant identified phases are shown at the top of the figure for [1] Quartz (SiO_2); [2] Mullite ($Al_{4.645}Si_{1.36}O_{9.68}$); [3] Hematite ($Fe_2O_3$); [4] Magnetite ($Fe_3O_4$); [5] unspecified zeolite 1; [6] unspecified zeolite 2 and [7] small angle scattering.

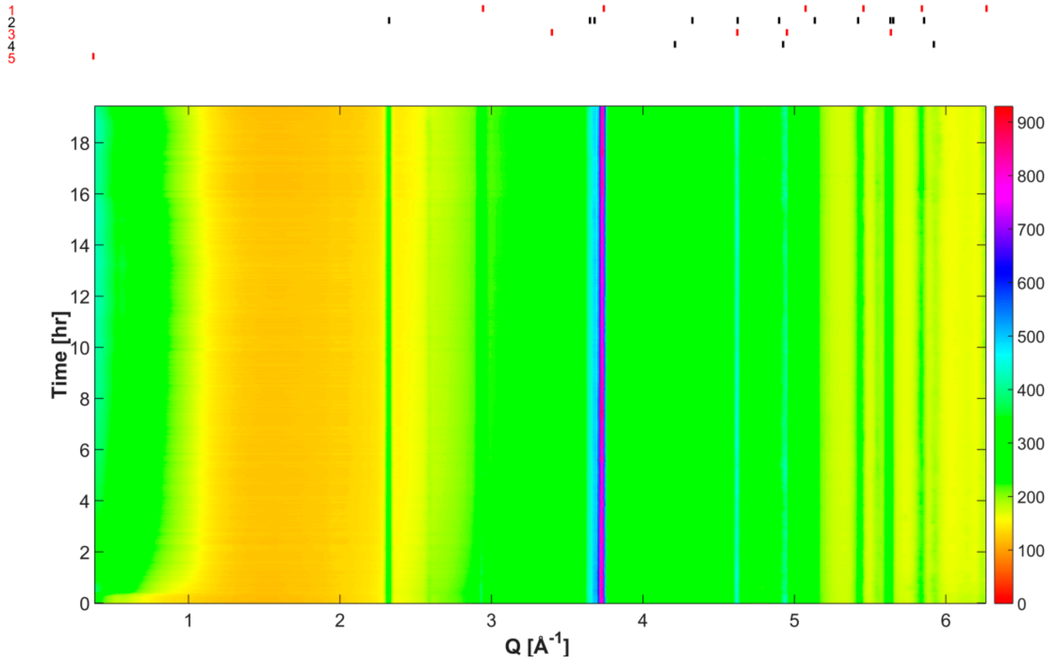

Figure S2. Time resolved diffraction pattern of CFA-2.0-0.8-5.5. Features include the evolution of low angle intensity (Q < 1 Å^{-1}) and the 'shift' in the amorphous peak position. The peak positions of significant identified phases are shown at the top of the figure for [1] Quartz (SiO_2); [2] Mullite ($Al_{4.645}Si_{1.36}O_{9.68}$); [3] Hematite ($Fe_2O_3$); [4] Magnetite ($Fe_3O_4$) and [5] small angle scattering.

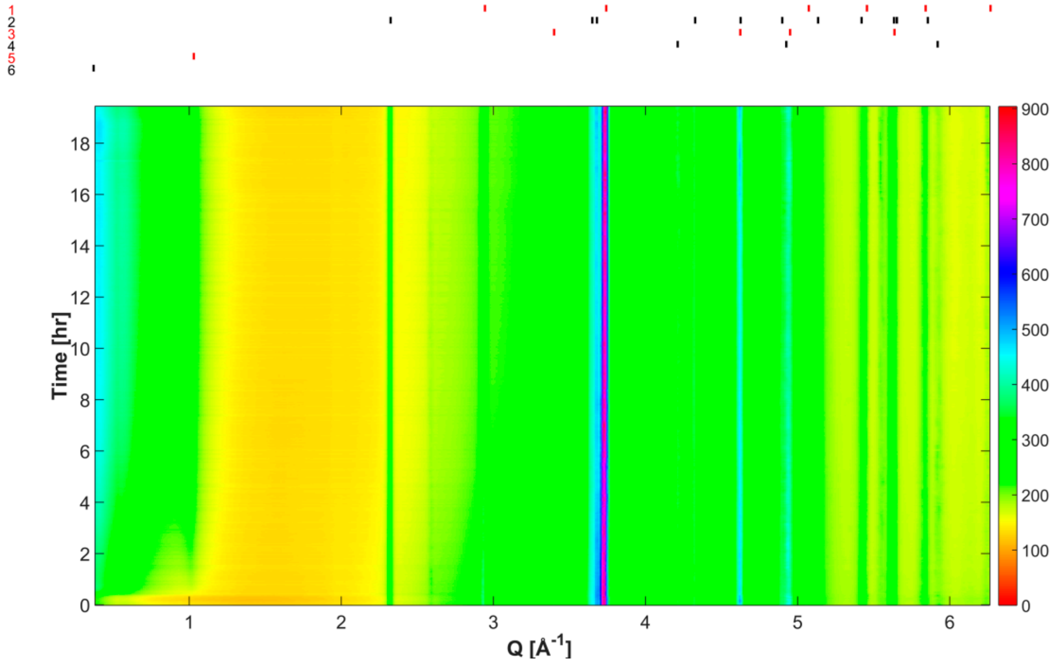

Figure S3. Time resolved diffraction pattern of CFA-2.0-1.2-5.5. Features include the evolution of low angle intensity (Q < 1 Å^{-1}), formation of a zeolite phase and the 'shift' in the amorphous peak position. The peak positions of significant identified phases are shown at the top of the figure for [1] Quartz (SiO_2); [2] Mullite ($Al_{4.645}Si_{1.36}O_{9.68}$); [3] Hematite ($Fe_2O_3$); [4] Magnetite ($Fe_3O_4$); [5] unspecified zeolite 3 and [6] small angle scattering.

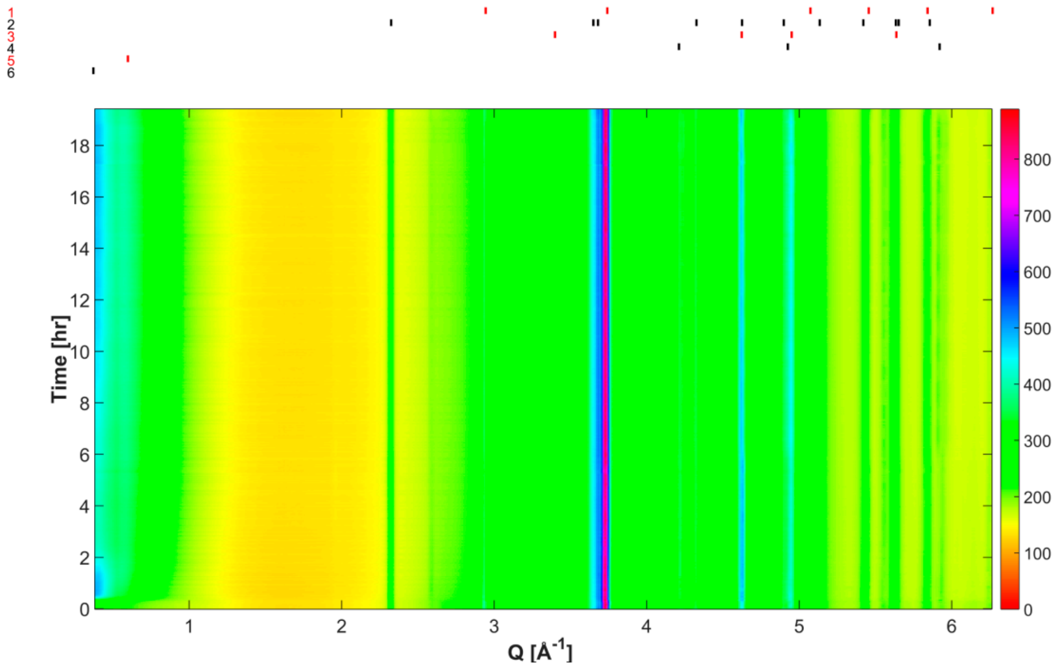

Figure S4. Time resolved diffraction pattern of CFA-2.0-0.8-5.5. Features include the evolution of low angle intensity (Q < 1 Å^{-1}), formation of a zeolite phase and the 'shift' in the amorphous peak position. The peak positions of significant identified phases are shown at the top of the figure for [1] Quartz (SiO_2); [2] Mullite ($Al_{4.645}Si_{1.36}O_{9.68}$); [3] Hematite ($Fe_2O_3$); [4] Magnetite ($Fe_3O_4$); [5] unspecified zeolite 4 and [6] small angle scattering.

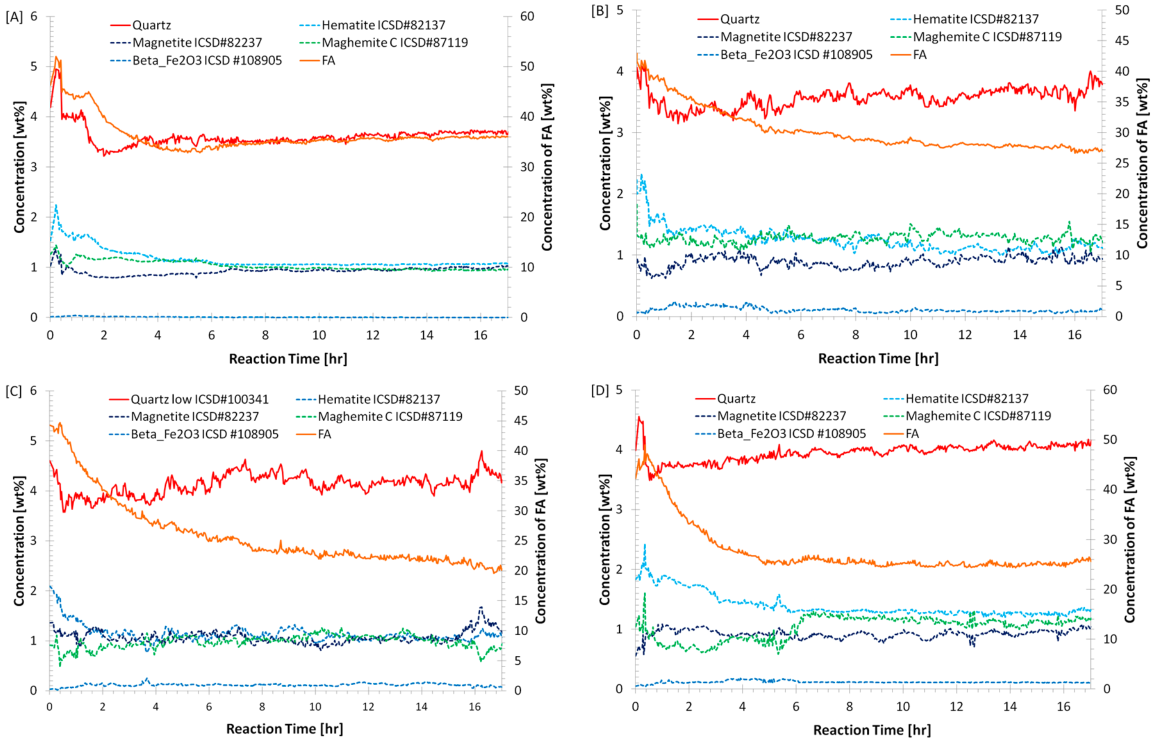

Figure S5. Quantitative phase results from the refinement (**A**) CFA-1.8-0.8-5.5; (**B**) CFA-2.0-1.2-5.5; (**C**) CFA-2.0-0.8-5.5; (**D**) CFA-2.2-0.8-5.5. The concentration of the flyash (FA) amorphous phase is shown on the right hand side axis.

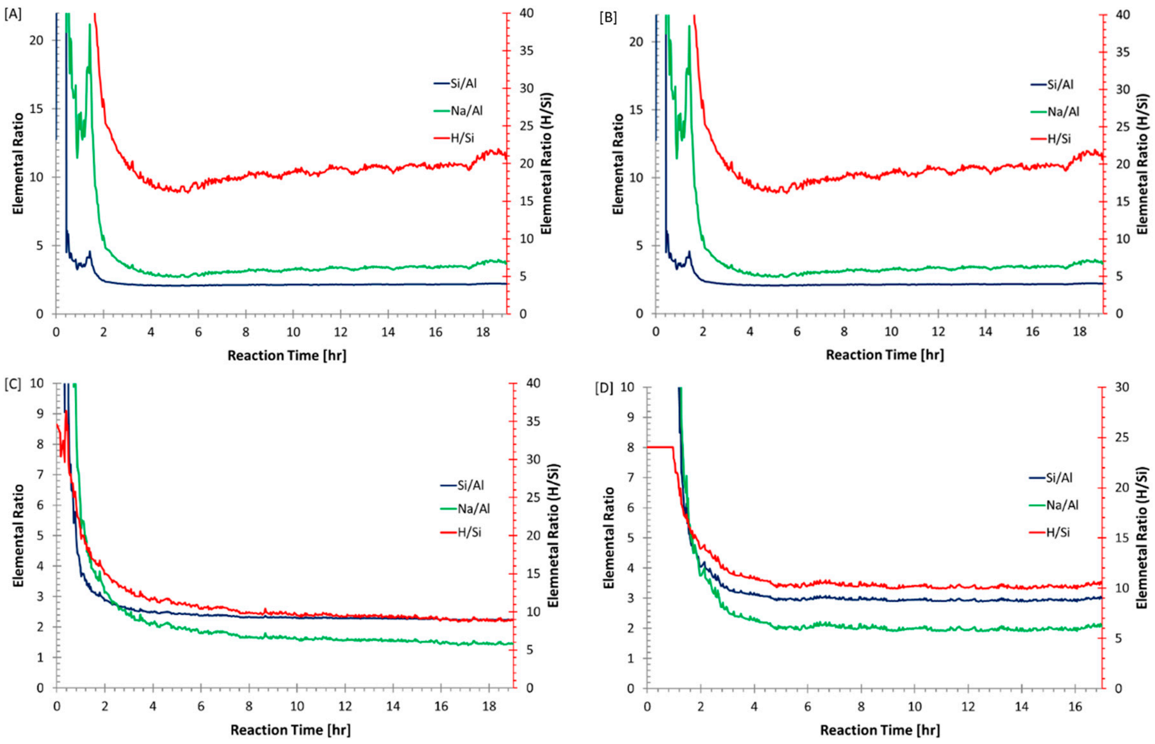

Figure S6. Elemental ratios of the reaction products as function of time (**A**) CFA-1.8-0.8-5.5; (**B**) CFA-2.0-1.2-5.5; (**C**) CFA-2.0-0.8-5.5; (**D**) CFA-2.2-0.8-5.5. Note: that the elemental ratio levels out 4–5 h into the reaction.
